# Supplementary material for: M1 Cholinergic Signaling Modulates Cytokine Levels and Splenocyte Sub-Phenotypes Following Cecal Ligation and Puncture
Source: Res Sq. 2023 Oct 5:rs.3.rs-3353062. Preprint. [Version 1] doi: 10.21203/rs.3.rs-3353062/v1 (PMC10602092; doi:10.21203/rs.3.rs-3353062/v1)
Supplement: Supplement 1 [file NIHPPRS3353062V1-supplement-1.pdf]

Supplementary Files

This is a list of supplementary files associated with this preprint. Click to download.

- M1AChRPaperSupplFig.pptx
- M1AChRSuppTable.pptx
